# Enriched Housing Reduces Disease Susceptibility to Co-Infection with Porcine Reproductive and Respiratory Virus (PRRSV) and *Actinobacillus pleuropneumoniae* (*A*. *pleuropneumoniae*) in Young Pigs

**DOI:** 10.1371/journal.pone.0161832

**Published:** 2016-09-08

**Authors:** Ingrid D. E. van Dixhoorn, Inonge Reimert, Jenny Middelkoop, J. Elizabeth Bolhuis, Henk J. Wisselink, Peter W. G. Groot Koerkamp, Bas Kemp, Norbert Stockhofe-Zurwieden

**Affiliations:** 1 Livestock Research, Wageningen University and Research Centre, Wageningen, the Netherlands; 2 Adaptation Physiology Group, Wageningen University and Research Centre, Wageningen, the Netherlands; 3 Central Veterinary Institute, Wageningen University and Research Centre, Lelystad, the Netherlands; 4 Farm Technology Group, Wageningen University and Research Centre, Wageningen, the Netherlands; University of Minnesota, UNITED STATES

## Abstract

Until today, anti-microbial drugs have been the therapy of choice to combat bacterial diseases. Resistance against antibiotics is of growing concern in man and animals. Stress, caused by demanding environmental conditions, can reduce immune protection in the host, influencing the onset and outcome of infectious diseases. Therefore psychoneuro-immunological intervention may prove to be a successful approach to diminish the impact of diseases and antibiotics use. This study was designed to investigate the effect of social and environmental enrichment on the impact of disease, referred to as “disease susceptibility”, in pigs using a co-infection model of PRRSV and *A*. *pleuropneumoniae*. Twenty-eight pigs were raised in four pens under barren conditions and twenty-eight other pigs were raised in four pens under enriched conditions. In the enriched pens a combination of established social and environmental enrichment factors were introduced. Two pens of the barren (BH) and two pens of the enriched housed (EH) pigs were infected with PRRSV followed by *A*. *pleuropneumoniae*, the other two pens in each housing treatment served as control groups. We tested if differences in disease susceptibility in terms of pathological and clinical outcome were related to the different housing regimes and if this was reflected in differences in behavioural and immunological states of the animals. Enriched housed pigs showed a faster clearance of viral PRRSV RNA in blood serum (p = 0.014) and histologically 2.8 fold less interstitial pneumonia signs in the lungs (p = 0.014). More barren housed than enriched housed pigs developed lesions in the lungs (OR = 19.2, p = 0.048) and the lesions in the barren housed pigs showed a higher total pathologic tissue damage score (p<0.001) than those in enriched housed pigs. EH pigs showed less stress-related behaviour and differed immunologically and clinically from BH pigs. We conclude that enriched housing management reduces disease susceptibility to co-infection of PRRSV and *A*. *pleuropneumoniae* in pigs. Enrichment positively influences behavioural state, immunological response and clinical outcome in pigs.

## Introduction

Psycho-social stress in man and animal alters disease susceptibility to infectious agents [1–4]. Social factors, environment and stress are associated with disease susceptibility and are therefore suggested as cofactors in the pathogenesis of infectious diseases [1, 5]. Antimicrobial drug therapy has been used against infectious bacterial diseases, but the resistance of bacteria to antibiotics is of increasing concern. Therefore psychoneuro-immunological intervention has been proposed as a potential strategy to diminish the onset and outcome of infectious diseases. However, many uncertainties remain regarding the effectiveness of this strategy [1].

In pigs, the adverse effects of stimulus-poor housing conditions and social stress on behaviour, (re)activity of the hypothalamic-pituitary-adrenal (HPA) axis and mood (pessimistic versus optimistic) are well established [6–20]. Provision of suitable enrichment substrates, such as straw or peat, better meet the behavioural needs of pigs and improve their welfare, but is often associated with disadvantages in costs, labour and hygiene and is incompatible with the generally applied slurry-based manure management [21]. Commercially kept pigs are therefore often reared under barren, stimulus-poor housing conditions [22]. Studies on the association between the use of natural materials, such as straw, as enrichment substrates and the risk of infectious diseases in pigs are equivocal [21, 23–26]. Pigs reared under enriched conditions exhibited fewer days of diarrhoea after weaning and less gastric lesions at slaughter as compared to barren reared pigs [6, 27–32]. No direct evidence has been established so far, however, for an effect of housing and management procedures that stimulate natural (social) behaviour in pigs on the development of disease, referred to in this paper as ‘disease susceptibility’ [5, 21, 33]. Therefore, in the current study we compared disease development following co-infection with porcine reproductive and respiratory syndrome virus (PRRSV) and *A*. *pleuropneumoniae* between pigs reared in environmentally and socially enriched pens and pigs reared in barren, stimulus-poor housing conditions. We hypothesized a reduced disease susceptibility to co-infection, as expressed by reduced clinical signs, in the enriched housed pigs.

We used this model of co-infection with mild virulent pathogens under experimental conditions to obtain measurable variation in clinical symptoms and pathology in the animals. When applied separately, these pathogens do not induce overt clinical signs or pathology, but in combination, probably due to PRRSV’s immunosuppressive effect, a higher incidence of secondary bacterial infections occurs [34]. Moreover, PRRSV and *A*. *pleuropneumoniae* are pathogens frequently involved in porcine respiratory disease complex (PRDC) in pigs. PRDC causes severe health and welfare problems, is difficult to control and due to morbidity and reduced growth rates in surviving animals incurs a significant financial burden to farmers. Although PRRSV vaccines have been developed, none of the current vaccines are able to completely prevent respiratory infection. Moreover, unfortunately current control strategies fail to provide sustainable disease control [35]. Enriched housing conditions may possibly contribute to sustainable disease control [34–36].

## Materials and Methods

The established principles of laboratory animal use and the EU and Dutch laws related to animal experiments were adhered to in this study. The Wageningen University Animal Care and Use Committee (Lelystad Department) approved the experiment under number 2013181.

### Experimental design, animals and housing

For the experiment 56 male and female piglets (Topigs 20 line, from Great Yorkshire x Landrace origin) were used (8 litters of 7 piglets). The piglets were offspring of eight multiparous sows obtained from a PRRSV and *A*. *pleuropneumoniae*-free herd. Sows were inseminated on the same day and the expected parturition day was defined as day 0 for all piglets. From two weeks before parturition onwards, the sows were housed in farrowing crates at research facility A of Wageningen UR Livestock Research, Lelystad, the Netherlands.

The 8 litters were subjected from the first day of life onwards to either barren housing (barren housed: BH) or enriched housing (enriched housed: EH) (Table 1). BH piglets were housed according to current legal requirements for farmed pigs in conventional 5 m^2^ barren pens with 100% slatted floor and a 100x45cm solid rubber floor mat. EH piglets were housed in 10m^2^ enriched pens with partly slatted (40%) and partly solid (60%) floor.

**Table 1:** Experimental set up and group names.

Housing	Groups before infections	Groups after infections	Number of pigs	Infection	Number of pens
Barren	BH	BHI	14	PRRSV / App	2
Barren	BH	BHC	7	Mock infected (Control)	1
Barren	BH	BHC	7	No infection (Control)	1
Enriched	EH	EHI	14	PRRSV / App	2
Enriched	EH	EHC	7	Mock infected (Control)	1
Enriched	EH	EHC	7	No infection (Control)	1

App: *A*. *pleuropneumoniae*

BH: Barren Housed, BHI: Barren Housed Infected, BHC: Barren Housed Control

EH: Enriched Housed, EHI: Enriched Housed Infected, EHC: Enriched Housed Control

In the barren pens two chains with blocks were added as enrichment. In the enriched pens rooting substrate was provided which consisted of 1 kg straw, 160 L of moist peat and 180 L of wood shavings. In these pens two jute bags and branches of a broom were added, as well as the same two chains with blocks as in the barren pens. Straw and wood shavings were replenished daily (0.5 kg/day straw, 23 L/day of wood shavings), and on a weekly basis new branches of a wooden broom, new jute bags and 20 L of peat were added to the enriched pens. All pens were cleaned daily by removing faeces and rinsing the slatted floors with water. All materials for all pens, including substrate enrichment and food, were γ-irradiated (9kGy irradiation; Synergy Health Ede BV, the Netherlands). A heating lamp for the piglets was provided in each pen during the first week after birth. On day 3, the piglets received an ear tag and they were treated with ironject^®^ 20% + B12 (Dopharma, Raamsdonksveer, the Netherlands) according to standard procedures. Tails were kept intact and the male piglets were not castrated.

All enriched and barren pens had two drinking nozzles, one for the sow and one for the piglets. Sows were fed a standard commercial diet twice a day at 8:00 am and 3:15 pm. The piglets received solid food *ad libitum*, starting at day 3. Lights were on between 6:00 am and 6:00 pm. Temperature was kept at 25°C during the first week after birth and it was decreased by 1°C every week until it reached 22°C, the week before weaning.

From day 13 until weaning, the panels between two adjacent EH pens were removed, allowing piglets from two different EH litters to mingle. Thus, the four individual enriched pens of 10m^2^ were temporarily transformed into two pens of 20m^2^ to enable early social interaction between EH litters.

On day 17 all piglets were subjected individually to the backtest, to assess their coping strategy [37]. During the backtest, piglets were held in supine position for 1 minute and the number of struggles, latency to first struggle, number of vocalizations, and latency to the first vocalization were recorded [38].

On day 31, the sows were removed from both barren and enriched pens (i.e. weaning day) and seven piglets per litter were selected. The selection was made taking gender, bodyweight and coping strategy into account in order to obtain piglets with comparable features in each experimental subgroup. The selected piglets were regrouped into eight new groups of seven piglets per group. All piglets were equally mixed and new groups with comparable compositions were formed. The new BH groups included only piglets originating from BH litters and the EH groups included only piglets originating from the EH litters, meaning that housing treatment remained the same as before weaning. The piglets per group were individually tagged using an animal marker for identification.

On day 39, all piglets were transported 4.5 km to facility B of Wageningen UR Livestock Research. Group structure, housing enrichment, pen sizes and access to food and water were kept the same as before transport. Pigs of two barren and two enriched pens were housed in separate High Efficiency Particulate Air (HEPA) filtered animal rooms to prevent pathogen distribution by air. The pigs of the other two barren and the two enriched pens served as negative-controls and were kept in conditions according to their housing conditions in separate pens within one large room in facility B without extra biosafety measures regarding the outgoing air (infected pigs referred to as BHI and EHI pigs, controls as BHC and EHC pigs, Table 1).

Temperature at weaning on day 31 was 28°C and was decreased by 2°C each week until it reached 22°C. It was kept at 22°C from day 42 to day 55.

On day 55 the pigs were euthanized by injecting pentobarbital (Euthasol40%, AST Farma) in the auricular vein, while they were restrained and thereafter exsanguinated.

### Behavioural observations and skin lesions

Frequencies of the behaviours listed in the ethogram (Table 2, adapted from [39, 40]) were recorded before and after weaning (day 30 and day 32) and before and after transport to facility B (day 38 and day 40). On each observation day, all pens were observed for the duration of 4 ten-minute periods, twice in the morning (between 8:00 and 11:00 am) and twice in the afternoon (between 13:00 and 16:00 am) in an order balanced for housing. Behaviours were averaged per pen and per day and expressed as frequencies per pig per ten minutes. A new bout was scored when the pig stopped the behaviour for more than 2 seconds.

**Table 2:** Ethogram.

Behaviour	Description
Social behaviour	Touching or sniffing any body part of a pen mate (frequently head region)
Aggression	Uni- or bilateral fighting by chasing, head knocking (with or without biting) and/or pushing
Manipulate pig	Nibbling, sucking or chewing on any body part of piglet or sow, or belly nosing
Manipulate pen	Nibbling, sucking or chewing on pen components
Playing	Fast running around the pen (galloping), rolling and shaking objects
Mounting	Standing on hind legs with front legs on pen mate

On the same days, skin lesions at the front (head, neck shoulders and front legs), middle (flanks and back) and rear (rump, hind legs, tail) were counted and categorized as a proxy for aggressive behaviour [41]. For each body region, the number and severity of lesions was differentiated using scores from 0–4 as follows (modified from [42]): **0**: No lesions; **1**: < 5 superficial lesions; **2**: 5–10 superficial lesions or < 5 deep lesions; **3**: 10–15 superficial lesions or 5–10 deep lesions; **4**: > 15 superficial lesions or > 10 deep lesions. Lesion scores were averaged per pen and per day.

### Infection procedure

On day 44 (from here on referred to as infection day 0: ID0), BHI and EHI pigs were inoculated with the mild-virulent European PRRSV serotype 1 strain LV-Ter Huurne. This PRRSV strain was isolated during the 1991 epizootic from a clinical case of PRRS in the Netherlands and was used at the 7^th^ passage on porcine alveolar macrophages (PAMs) [43]. Pigs were infected intranasally with 1.5 ml inoculum containing 5 log_10_ 50% tissue culture infectious dose (TCID_50_). This treatment was followed by an aerosol infection at day 52 (ID8) with *A*. *pleuropneumoniae* serotype 2. The inoculum of *A*. *pleuropneumoniae* serotype 2 strain 17415 (reference strain) was prepared as described earlier [44]. In short, an aliquot of the reference strain, which was stored at –70°C, was thawed and the suspension was cultured overnight at 37°C under microaerophilic conditions on sheep blood agar plates (SB plates) with nicotinamide adenine dinucleotide (NAD). The next morning, colonies were transferred to fresh SB + NAD plates and cultured for 6 hours. Thereafter colonies were rinsed from the plates and stored in phosphate buffer saline (PBS-13). The number of colony forming units (CFU) in the suspension was determined overnight and the inoculum diluted to achieve a concentration of 9 log_10_ CFU *A*. *pleuropneumoniae*/ml suspension. Groups of two to three pigs were simultaneously exposed in a stainless steel aerosol chamber (110x 45 x 90 cm). Aerosol was administered using the aerosol nebulizer Aeroneb Pro (EMKA Technologies, Paris, France). An amount of 5 ml of the inoculum suspension was administered during a period of 20 min. The procedures have previously been described [45, 46].

Half of the BHC and EHC pigs were not inoculated (negative controls) and underwent no extra handlings. The other half of the BHC and EHC pigs underwent the same procedures as the infected animals (mock control) at ID0 and ID8 (Table 1), however instead of PRRSV inoculum, 1.5 ml RPMI medium was used and instead of the *A*. *pleuropneumoniae* inoculum, 5 ml of PBS was used.

### Sickness behaviour

Cameras were placed in all pens in facility B. On day ID-1, ID3, ID6 and ID9, postures and behavioural activities of pigs were scored with 10-min instantaneous scan sampling from 7:00 am until 6:00 pm using the Observer XT 10.0 resulting in 66 scans per pig per day (Noldus Information Technology B.V., Wageningen, the Netherlands). From these observations, both ‘lying behaviour’ (i.e. proportion of scans lying), ‘behavioural activity’ (i.e. proportion of scans during which pigs showed active behaviours, such as eating, drinking, social behaviours, manipulation of pen or pen mates and aggression) and ‘drinking and eating behaviour’ (i.e. proportion of scans during which pigs were (apparently) drinking and eating) were calculated.

### Rectal temperature, clinical examination, growth

Rectal temperature was assessed once at ID-2 and from ID0 until ID11 twice per day (first measurement between 8–11 am and second measurement between 3–4 pm, referred to as IDX(1) and IDX(2), respectively). At the days of infections, temperature was measured prior to the infection procedure and exactly 4 hours after the infection procedure took place. Microlife digital thermometers with a resolution of 0.1°C were used. Thermometers were calibrated prior to the experiment.

All piglets were observed and inspected for respiratory symptoms (coughing, breathing problems, sneezing) twice per day (at 9:00 am and 4:00 pm) from ID0 until the end of the experiment (ID11). Body weight of the piglets was measured weekly from the day of parturition until the end of the experiment. Average daily growth was calculated from ID0 to ID8 for the period after PRRSV and from ID8 to ID12 for the period after *A*. *pleuropneumoniae* infection.

### Phenotyping of white blood cells and broncho-alveolar cell populations

Blood samples (serum, EDTA blood) were taken by jugular vein puncture on 7 different days (ID0, ID2, ID4, ID8, ID9, ID10 and ID11). The total count of white blood cells (WBC) and a differential count of lymphocytes, granulocytes and monocytes were performed with a haematology analyser (blood cell counter Sysmex pocH-100iV diff, Kobe, Japan).

Freshly isolated heparinized blood samples (100μl) were used to quantify different phenotypes of WBC’s by flow cytometry (FCM). Briefly, the blood cells were incubated with a primary antibody-mixture of monoclonal antibodies (mAb) against CD3, CD4, CD8 for triple labelling or for single labelling with mAb against CD172a or CD21 (Table 3). The following non-related isotype controls (Southern Biotech) were used for the different Ig classes: IgG1 (clone 15H6), IgG2a (clone HOPC-1), IgG2b (clone A-1) and IgM (clone 11E10).

**Table 3:** Antibody panels for FACS.

	Antigen	Clone	Isotype	Fluorochrome	Labelling	Source of antibody
**White blood cells**	*Triple staining*					
	CD3	PPT3	IgG1	APC	Secondary^1^	Southern Biotech
	CD4	74-12-4	IgG2b	FITC	Secondary^1^	Southern Biotech
	CD8α	76-2-11	IgG2a	PE	Secondary^1^	Southern Biotech
	*Single staining*					
	CD172a	74-22-15	IgG2b	FITC	Secondary^1^	VMRD
	CD21	B6-11C9	IgG1	APC	Secondary^1^	Southern Biotech
**BALF cells**	*Triple staining*					
	CD3	PPT3	IgG1	APC	Secondary^1^	Southern Biotech
	CD4	74-12-4	IgG2b	FITC	Secondary^1^	Southern Biotech
	CD8α	76-2-11	IgG2a	PE	Secondary^1^	Southern Biotech
	*Double staining*					
	CD172a	74-22-15	IgG2b	PE	Secondary^1^	VMRD
	CD172a	74-22-15	IgG1	PE	Secondary^1^	Beckman Coulter
	CD 14	MIL2	IgG2b	FITC	Primary	BioSource
	SLAII-DR	2E9/13	IgG2b	FITC	Primary	Serotec
	CD163	2a10/11	IgG1	FITC	Primary	Serotec
	TLR4		IgM	FITC	Secondary^1^	Gift by J. Dominguez
	*Single staining*					
	Granulocytes	2B2	IgG1	FITC		Serotec

^1^: For secondary labelling cells were incubated with isotype specific secondary antibodies (goat anti–mouse, source Southern Biotech)

In the next step, FACS™ Lysing Solution (BD Biosciences) was added for 10 minutes. After washing of cells in PBS supplemented with 1% (v/v) pig serum and 0.1% (w/v) sodium azide, cells were incubated with isotype specific secondary antibodies (goat anti–mouse IgG1-APC, IgG2b-FITC and IgG2a-PE, source Southern Biotech).

For the phenotyping of the alveolar cell population, broncho-alveolar lavage fluid (BALF) was obtained from the right cranial lung lobe during necropsy. A catheter was inserted in the local bronchus and the lobe was flushed with 30 ml PBS and ca. 15 ml inserted PBS was harvested. Cells in the BALF were immediately isolated and preserved as earlier described [47]. For the phenotypic characterization of intra alveolar lymphocytes, granulocytes and monocyte/macrophages, BALF cells were thawed, transferred to micro titre plates and either triple stained with mAb directed towards CD3, CD4, CD8a to quantify the various lymphocytic phenotypes or double stained with mAb against CD172a in combination with mAb against either CD14 or SLAII or CD163 or TLR4^+^ or single stained with mAb against porcine granulocytes (Table 3).

Flow cytometer analyses were performed with the FACS flow cytometer (Cyan ADP, Beckman Coulter) and evaluated with the Cyan ADP Summit 4.3 software. Blood cells were first gated on the basis of forward-scatter (FCS) versus sideward scatter (SSC) diagram as described [48] and then analysed according to their antigen marker profile. Based on the WBC counts in blood by the automated blood cell counter the absolute numbers of the different phenotypes were assessed. Granulocytes were identified in blood by an SSC^high^ CD172a^+^ profile and in BALF by the use of the granulocyte marker. T-cells were distinguished in the lymphocyte gate by the following marker combinations: naïve/non-activated T-helper cells: CD3^+^CD4^+^CD8^-^, cytotoxic T-cells: CD3^+^CD4^-^CD8^+^, memory/activated T-helper cells: CD3^+^ CD4^+^CD8^+^ and natural killer (NK) cells: CD3^-^CD4^-^CD8^+^ [48, 49]. Monocytes were identified in blood by low granularity, i.e. SSC^low^CD172a^+^. Macrophages in BALF were analysed by their CD172a^+^ (and CD14) expression in combination with the co-expression and expression levels, of their differentiating markers, i.e. mean fluorescence intensity (MFI). The alveolar cell populations are presented as percentage of the total cell population in the BALF. The macrophage markers are presented as percentage of total cell population in the BALF and as MFI.

### Detection of viral RNA in serum

A quantitative reverse transcription polymerase chain reaction (qRT-PCR) was performed on RNA isolated from blood serum, sampled at ID0, ID4, ID8, ID10 and ID11. A one-tube qRT-PCR was performed with the Applied Biosystem 7500 Fast System instrument using the Quantitect Probe RT-PCR kit from Qiagen. The reaction mixture (25 μl) contained 0.25 μl of kit-supplied enzyme, 12.5 μl of Quantitect Mix, 15 μM of each primer (Fw: 5’- GAT GAC RTC CGG CAY C -3’, Rev: 5’- CAG TTC CTG CGC CTT GAT -3’) and 10μM of probe (5’- Fam-TGC AAT CGA TCC AGA CGG CTT-Tamra- 3’)[50]. RT-PCR was performed at 30 min at 50°C and 15 min at 95°C followed by a two-step cycling protocol: 94°C for 20s, and 55°C for 45s for 40 cycles. Analysis was performed with 7500 Software v2.0.6 (Applied Biosystems). Viral RNA concentration (expressed as TCID_50_ equivalents per g) of each serum sample was calculated using a standard curve, constructed by extracting RNA from five decimal dilutions of medium spiked with known concentrations of infectious virus [51].

### Detection of typical *A*. *pleuropneumoniae* lesions and histological assessment of the lungs

After the pigs had been euthanized, special attention was paid during necropsy to patho-morphological changes in the respiratory tract. The type and size of lung lesions were recorded in a lung drawing and the proportions of the affected lung surface was calculated. Necro-haemorrhagic lesions, typical for *A*. *pleuropneumoniae*, were macroscopically detected and histologically confirmed. Total number of pigs with these lesions, were counted and presented as total number and percentage of pigs with typical *A*. *pleuropneumoniae* lesions.

For histological assessment of the lungs, 6 tissue samples per pig from predefined locations in the lungs (cranial, cardial and caudal lobe of the left and right lobe) were formalin-fixed, processed and embedded in paraffin. Tissue sections were stained with Haematoxylin-Eosin (HE) and a semi-quantitative, patho-histological assessment of HE stained slides encompassed the extent of pneumonia throughout the predefined locations in the lungs. Patho-histological assessment included 4 features: (1) the presence of focal or diffuse alterations with interstitial or catarrhal pneumonia or atelectasis, (2) the extent of infiltration of alveolar septae with mononuclear cells (3) the extent of infiltration of mononuclear cells in the perivascular/peribronchiolar area and (4) pleuritis. A histological score of 0 to 3 was used to describe the severity of changes per feature (i.e. 0 = no findings, 1 = mild focal manifestation, 2 = moderate, multifocal manifestation or diffuse manifestation, 3 = severe diffuse manifestation). During histological examination, the pathologist was blinded with regard to treatment (Housing and Infection). The scores from all 6 slides per lung were added to obtain an *overall histology score*, which could add up to a maximum of 72 points per pig (6 slides per pig, 4 histological features, and maximum score of 3 per feature). The *total interstitial components score* per pig was also calculated and presented separately as these changes in the lungs are considered to be related to PRRSV infection [52–55]. This score could reach a maximum of 18 (6 slides per lung, 1 histological feature, and maximum score of 3).

### Re-isolation of *A*. *pleuropneumoniae*

For re-isolation of *A*. *pleuropneumoniae*, two lung tissue samples of predefined lungs locations (cranial lobe, caudal dorsal lobe) and samples of bronchial lymph nodes were taken. When a macroscopic visible lung lesion was present at another location than the previous described locations, a third sample was taken. The collected lung and bronchial lymph node tissues were prepared, grinded and plated on SB+NAD plates as described by [56, 57]. To confirm that colonies were *A*. *pleuropneumoniae* serotype 2, dependency for NAD was tested by comparing growth, on SB and SB+NAD plates followed by agglutination with serotype 2-specific hyper-immune rabbit antiserum [57].

### Statistical analysis

All statistical analyses were performed with SAS (SAS 9.3, SAS Institute Inc.). The experiment was primarily designed to test the effect of housing management (enriched versus barren) on disease susceptibility. The mock-infected controls were used to make sure that the procedures to infect the pigs alone did not cause any differences. Preliminary statistical analysis (general linear model, GLM) showed indeed no differences in outcome (pathology, rectal temperature, growth and BALF variables) between mock-infected and negative control pigs. These groups were therefore combined and referred to as ‘control group’.

For all data, except the occurrence of lung lesions (see below), mixed linear models were used. Behaviour and skin lesions were analysed with pen as observational unit as behaviours and skin lesions of individual pigs within a group are not independent. All other variables were analysed with pig nested in the housing regime as observational unit.

Differences between variables at a specific time point (i.e. behaviour and skin lesions at the day before weaning, growth, histology scores and BALF cells) were analysed with housing and if applicable with infection and the interaction between housing and infection as fixed effect(s) and sow as random effect.

Differences between time dependent variables (i.e. behaviour and skin lesions after weaning, sickness behaviour, viral RNA, WBC, rectal temperature) were analysed with day as repeated effect using the repeated statement of SAS (SAS 9.3, SAS Institute Inc.) with the autoregressive covariance structure. Day, housing and if applicable infection and their interactions were used as fixed effects and sow as random effect.

Effects of housing on behaviour and skin lesions were analysed separately for the day before weaning, because pigs were mixed into other groups after weaning leading to a different group composition for the day after weaning, the day before transport and the day after transport. If needed, variables were square root transformed to obtain normally distributed residuals. In case the interaction effect for a variable was significant, post-hoc testing with Bonferroni adjustment was used.

Differences between BHI and EHI pigs in the occurrence of typical *A*. *pleuropneumoniae* lung lesions (i.e. lung lesions present or absent) and in occurrence of re-isolated *A*. *pleuropneumoniae* were analysed with a generalized mixed linear model (GLIMMIX procedure in SAS), with a logit link and binary distribution with housing as fixed effect and sow as random effect. Here, odds ratios (OR) are presented to indicate the extent of the effect.

## Results

### Behaviour and skin lesions before co-infection

**Fig 1:**
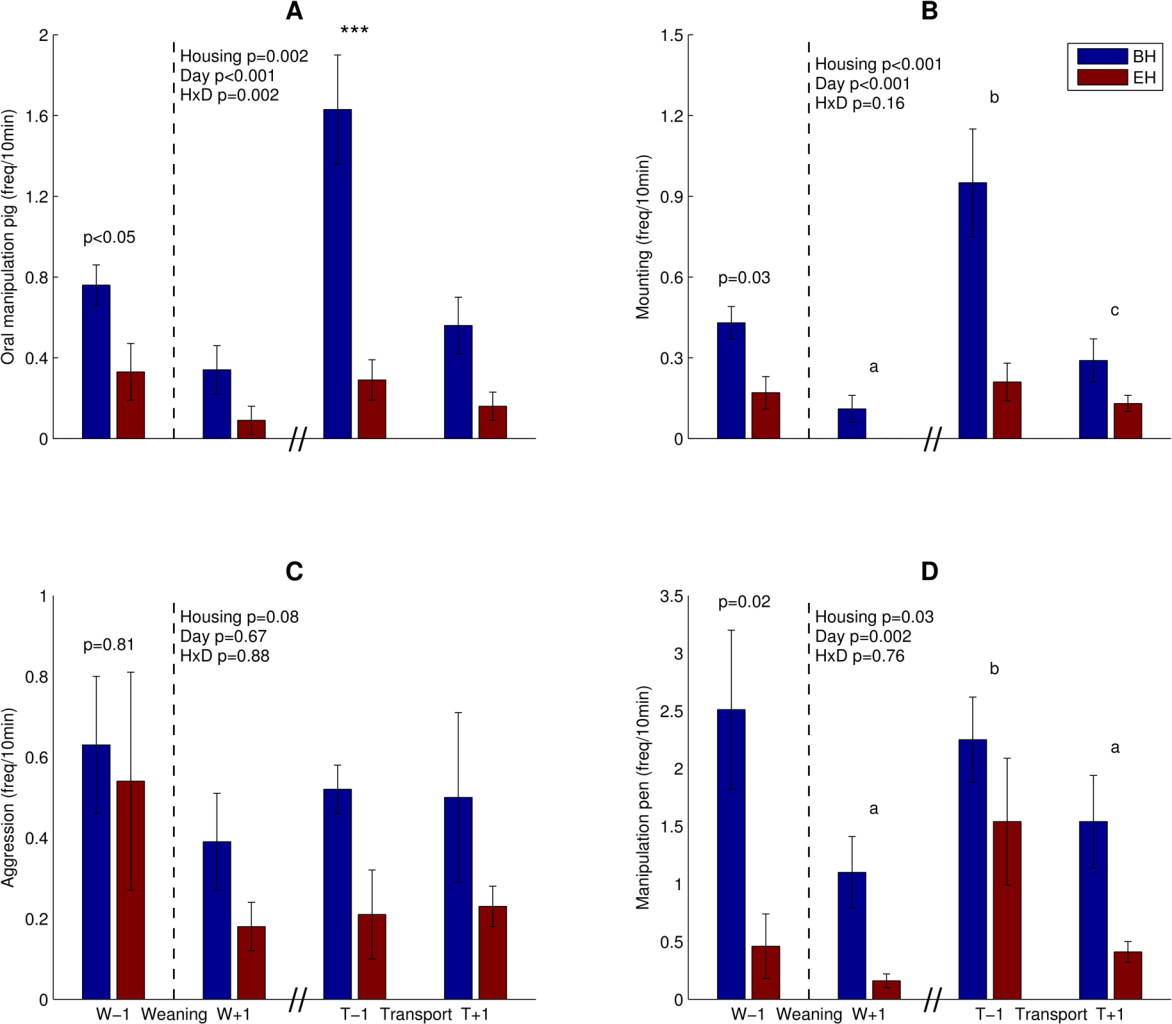
Behaviour of BH and EH pigs (frequencies/pig/10min, mean ± standard error). (A) Oral manipulation. (B) Mounting. (C) Aggression. (D) Manipulation pen. Blue bars: BH pigs, Red bars: EH pigs. W-1 and W+1: days before and after weaning. T-1 and T+1: days before and after transport. Black dotted vertical line indicates the separate statistical analysis of the day before weaning. HxD: housing-day interaction effect. Day effects are indicated as ‘a’-‘c’, bars with no common superscript differ significantly (p<0.05). (A) Post hoc comparison of housing effect at T-1, ***: p<0.001.

Before weaning, BH pigs showed 2.3 times more oral manipulation of other pigs (i.e. sow and piglets), 2.5 times more mounting and 5.4 times more manipulation of pen fixtures than enriched housed (EH) pigs (Fig 1A, 1B and 1D). Aggression (Fig 1C), social behaviour (0.61±0.07 vs. 0.56±0.13 times/pig/10 min, p = 0.72) and playing (0.35±0.18 vs. 0.50±0.20 times/pig/10 min, p = 0.59) did not differ between BH and EH pigs before weaning.

After weaning, oral manipulation was affected by housing, day and their interaction (Fig 1A). Overall BH pigs showed more oral manipulation as compared to EH pigs. Post hoc analysis revealed that BH pigs showed a 5.6 fold higher frequency of oral manipulation on the day before transport (Fig 1A). Mounting was also affected by housing, with higher frequencies for BH than for EH pigs, and day (Fig 1B). Pen manipulation was affected by housing with higher frequencies for BH than for EH pigs, and day (Fig 1D). Social behaviour was affected by day (p = 0.02), but not by housing (BH: 0.19±0.05 vs. EH: 0.15±0.03 times/pig/10 min, p = 0.41) or the interaction (p = 0.32). Housing (p = 0.60) and day (p = 0.18) did not significantly affect playing behaviour (BH: 0.18±0.09 vs. EH: 0.13±0.05 times/pig/10 min).

**Fig 2:**
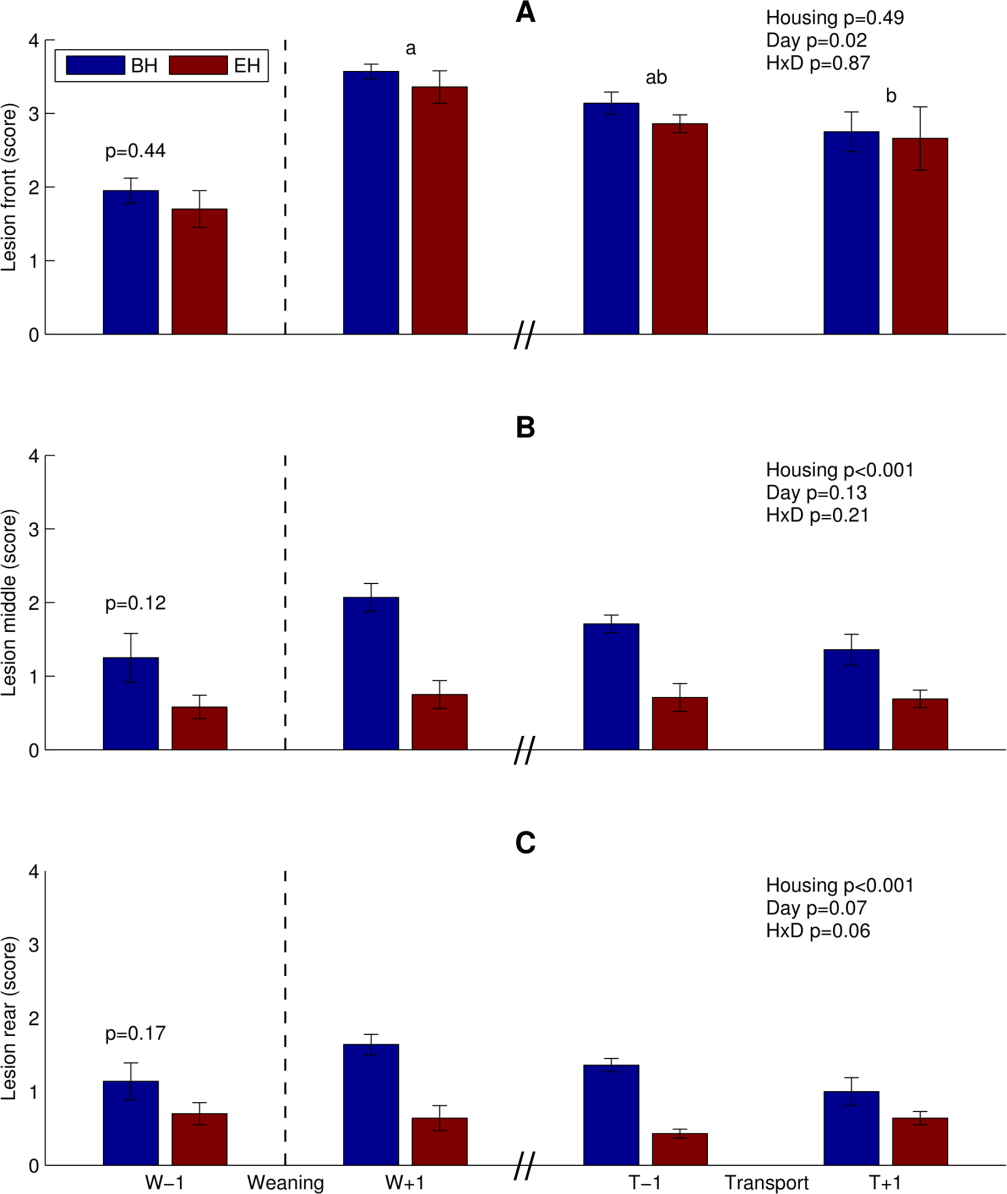
Skin lesion scores (mean of a four-point scale ± standard error). (A) Front. (B) Middle. (C) Rear of the body. Blue bars: BH pigs, Red bars: EH pigs. W-1 and W+1: days before and after weaning. T-1 and T+1: days before and after transport. Black dotted vertical line indicates the separate statistical analysis of the day before weaning. HxD: housing-day interaction effect. Day effects are indicated as ‘a’ and ‘b’, bars with no common superscript differ significantly (p<0.05).

Before weaning, skin lesion scores did not significantly differ between BH and EH pigs (Fig 2). After weaning, skin lesion scores on the front of the body were only affected by day (Fig 2A). BH pigs had more skin lesions on the middle and rear of their bodies after weaning than EH pigs (Fig 2B and 2C).

### Viral RNA in serum

**Fig 3:**
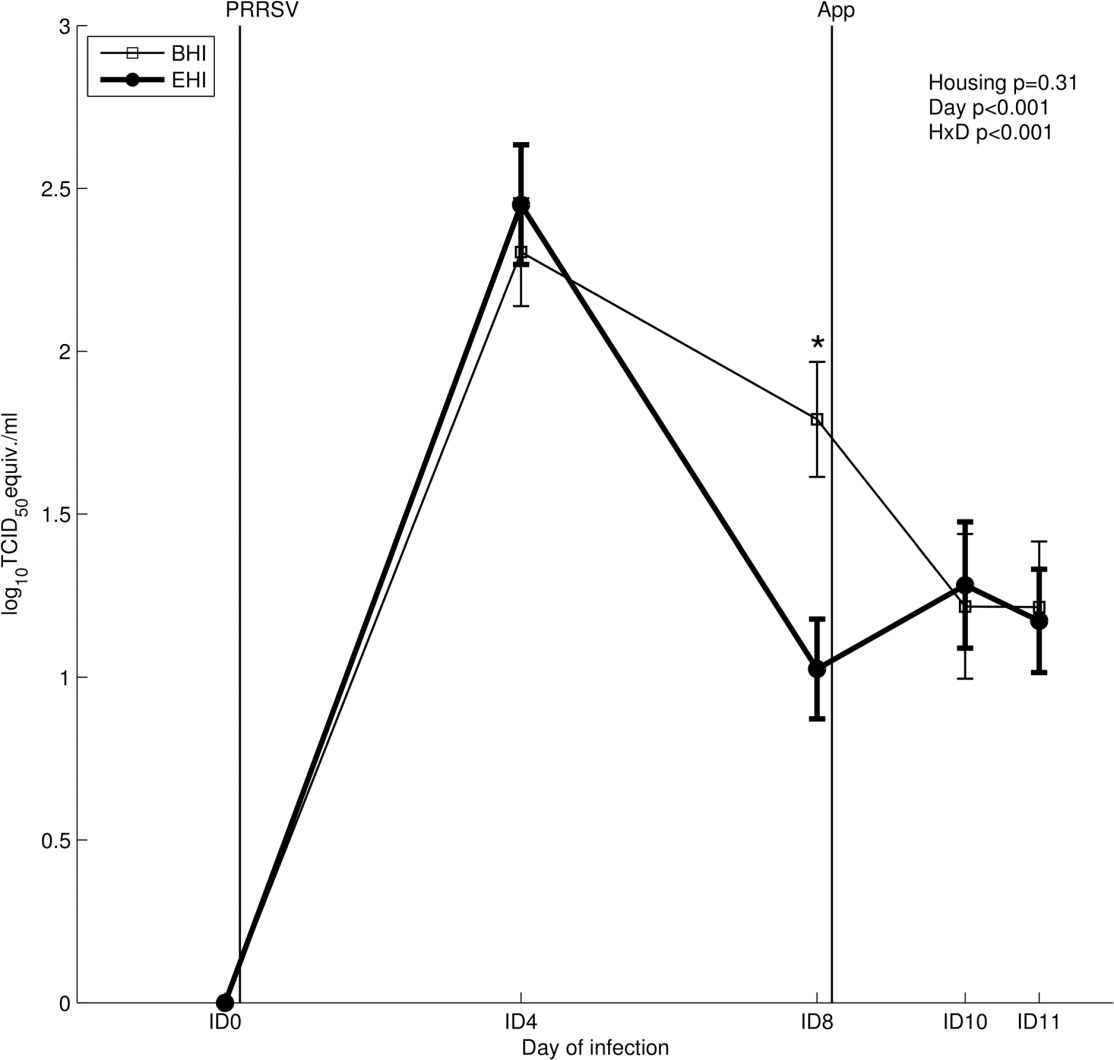
PRRSV qRT-PCR in serum of BHI and EHI pigs (means ± standard error). Infections at ID0 and ID8 with PRRSV and *A*. *pleuropneumoniae* are indicated as black vertical lines. HxD: housing-day interaction effect.* Post hoc comparison of housing effect at ID8 (p<0.05).

Viral RNA expressed as log10 TCID_50_ eq/ml was affected by day and the housing x day interaction (Fig 3). Post hoc analysis revealed that 4 days after PRRSV infection (ID4) viral RNA amount was equally increased in both BHI and EHI pigs as compared to day ID0, whereas at ID8 viral RNA was significantly lower in EHI pigs as compared to BHI pigs (Fig 3). No viral load differences were found between the two groups at ID10 and ID11 (Fig 3).

### Lung lesions

The most remarkable result was the impact of housing treatment on the number of pigs with typical *A*. *pleuropneumoniae* lesions. These lesions were characterized by a (multi)focal necro-haemorrhagic pleuro-pneumonia, which was macroscopically observed and histologically confirmed. More BHI pigs (8 out of 14, 57%) than EHI pigs (1 out of 14, 7.1%) developed lesions (OR = 19.2, p = 0.048). In 5 BHI pigs and 1 EHI pig, *A*. *pleuropneumoniae* was re-isolated successfully, but this difference was not significant (p>0.10).

**Fig 4:**
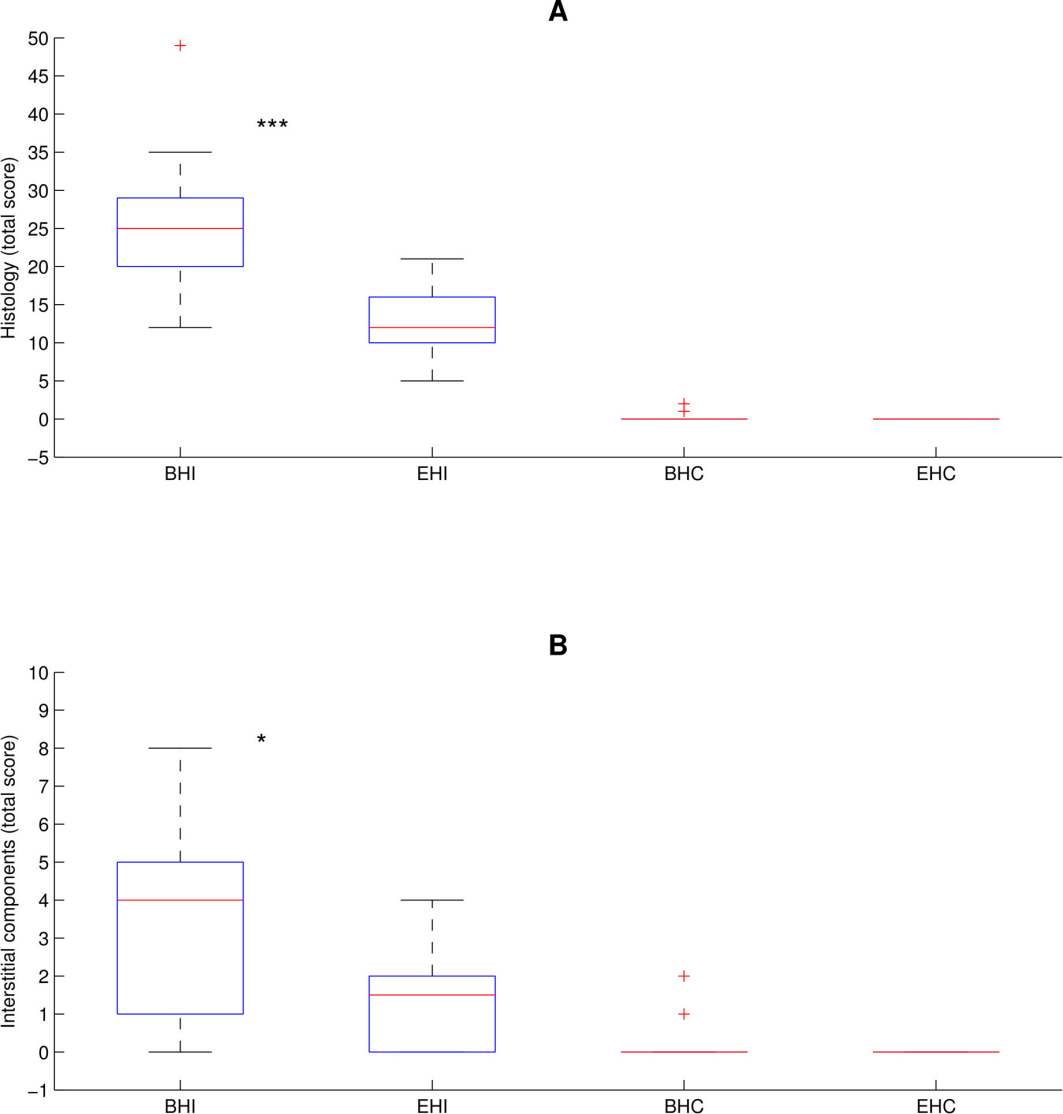
**Overall histology (A) and total interstitial component (B) score.** The central blue box indicates the lower and upper boundary at the 25% / 75% quantile of the data. The central red line indicates the median of the data. Two vertical lines extending from the central box indicate the remaining data outside the central box that are not regarded as outliers. Outliers are indicated as red crosses. ***: p<0.001; *: p<0.05.

To further address the extent of co-infection on lung morphology a broader histological evaluation of the lung samples was performed. In BHI pigs the patho-histological lung tissue score (i.e. the overall histological score) was significantly higher (2.1 fold, Fig 4A), meaning that the extension of altered tissue was larger and that more catarrhal-purulent exudates in alveoli, fibrinous or necro-haemorrhagic changes and fibrinous pleuritis was seen as compared to EHI pigs. Also, interstitial changes (i.e. the total interstitial components score) with increased peribronchiolar and perivascular lympho-monocytic cell infiltration were significantly increased in lungs of BHI pigs compared to EHI pigs (2.8 fold, Fig 4B).

### Sickness behaviour

**Fig 5:**
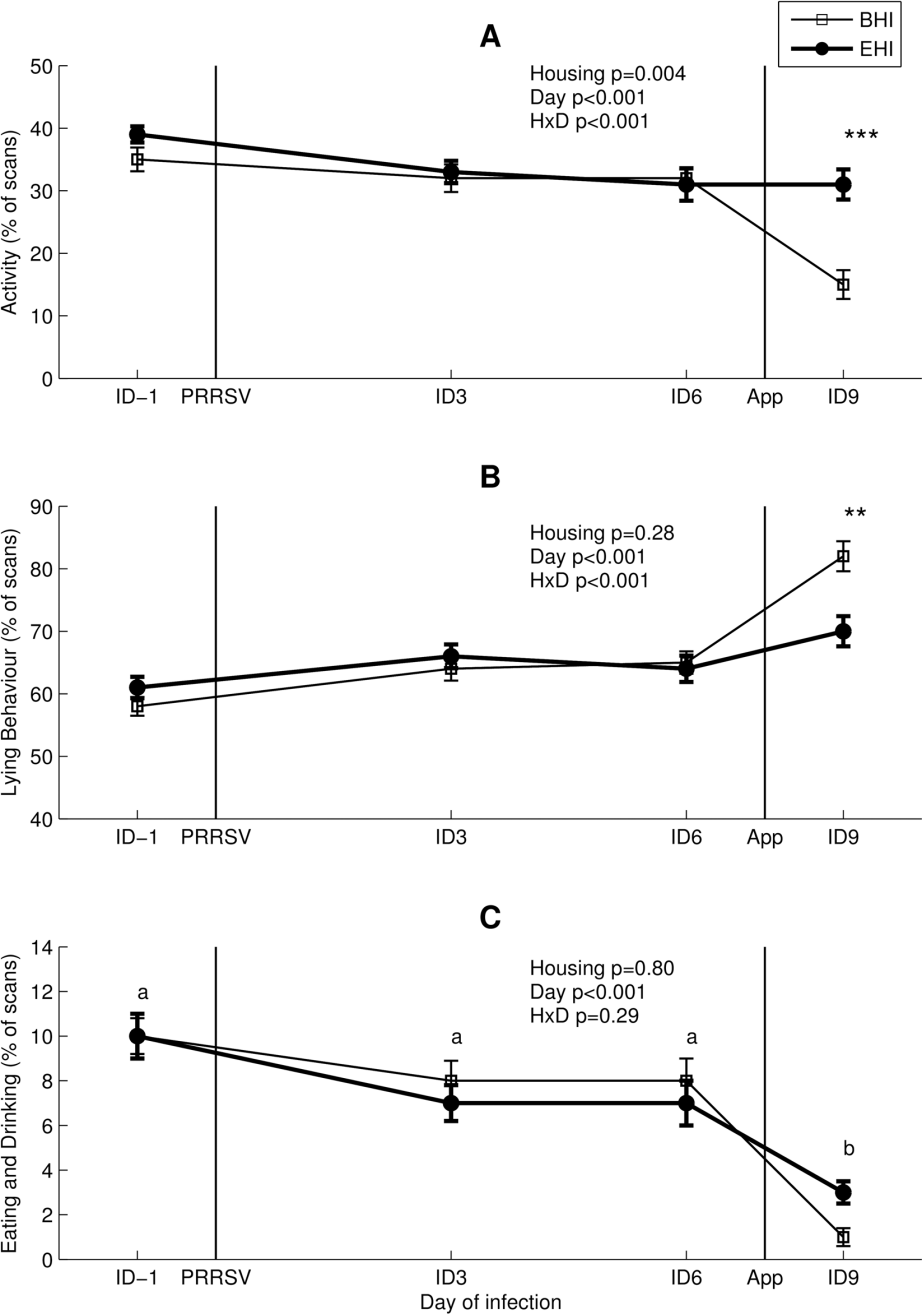
Sickness behaviour (mean (% of scans) ± standard error) of BHI and EHI pigs. (A) Activity. (B) Lying behaviour. (C) Eating and drinking. PRRSV (ID0) and *A*. *pleuropneumoniae* (ID8) infections are indicated as black vertical lines. HxD: housing-day interaction effect. Day effects are indicated as ‘a’ and ‘b’, days with no common superscript differ significantly (C) (p<0.05). Post hoc comparison at ID9, ***: p<0.001 (A), **: p<0.01 (B).

During daily inspections throughout the experiment, no coughing or breathing problems in any of the groups were detected that would have indicated the occurrence of respiratory problems. Sickness behaviour was addressed by analysing the animals’ active, lying and eating and drinking behaviour. An overall housing effect was seen for activity, but not for lying levels or eating and drinking behaviour as shown in Fig 5A–5C. A housing-day interaction was seen in activity and lying behaviour, but not for eating and drinking behaviour. A day effect was seen in all behaviours (Fig 5A–5C), indicating the difference in behaviour at ID9 as compared to the other days. Post hoc comparisons revealed that BHI pigs were less active, and spent more time lying than EHI pigs the day after *A*. *pleuropneumoniae* infection (ID9) (Fig 5A and 5B). All pigs spent less time visiting the drinker and feed trough the day after *A*. *pleuropneumoniae* infection (ID9) as compared to the other three days, as shown in Fig 5C.

### Rectal temperature and growth

**Fig 6:**
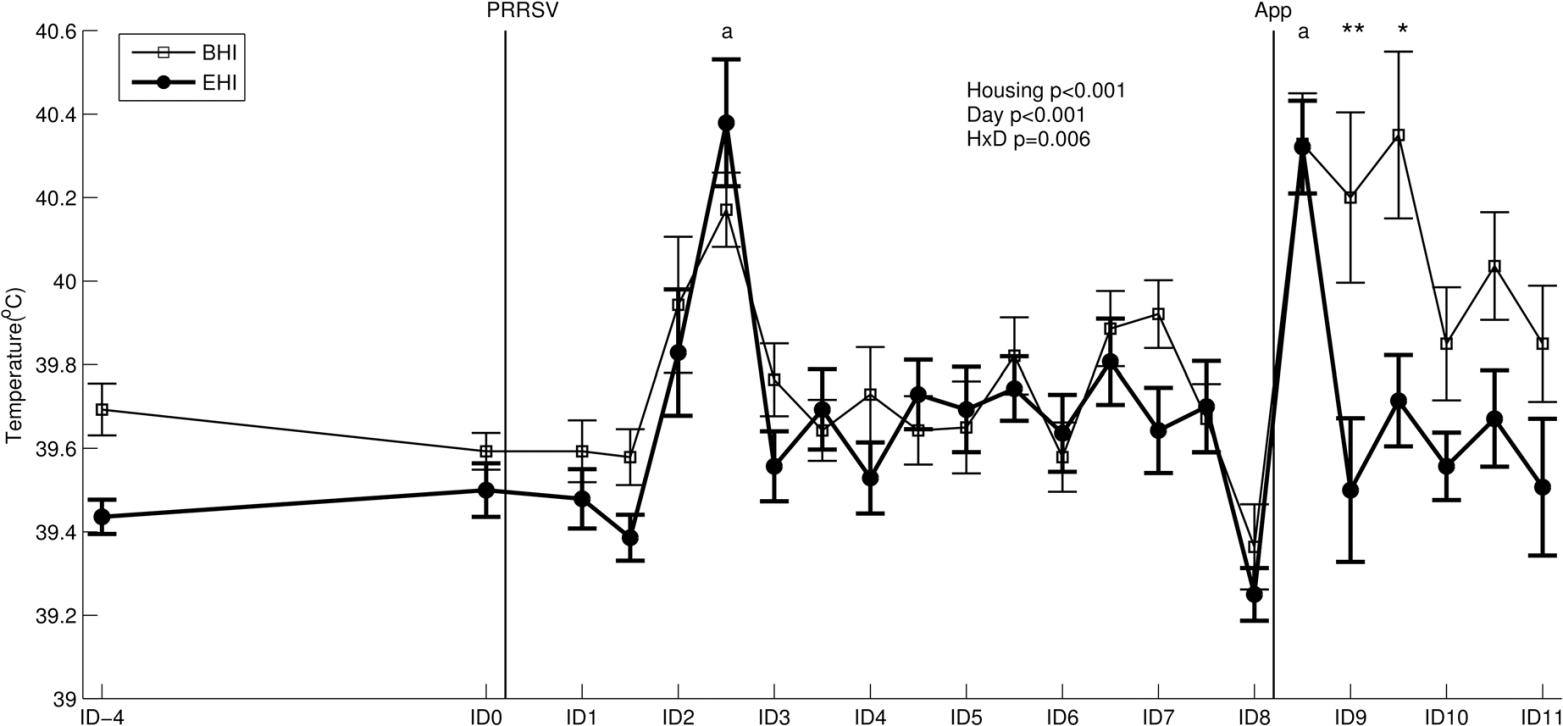
Rectal temperature (mean ± standard error) of BHI and EHI pigs. Days of infection (ID0 and ID8) are indicated as black vertical lines. HxD: housing-day interaction effect. ‘a’ denotes higher temperatures on ID2(2) and ID8(2) as compared with other days (both p<0.001). Post hoc comparison at ID9(1) and ID9(2): **: p<0.01 and *: p<0.05.

Housing, day and their interaction affected rectal temperatures of the infected pigs (Fig 6). Post hoc comparisons showed a rise in rectal temperature (of 0.7°C on average) after PRRSV infection in both BHI and EHI pigs (average 39.5°C at ID0 to 40.2°C at ID2(2), Fig 6). Average rectal temperature decreased again at ID3 in both groups similarly. After *A*. *pleuropneumoniae* infection (at ID8(1)) again both BHI and EHI pigs showed a similar peak temperature at 4 hours after the infection (ID8(2)). In BHI pigs rectal temperature remained raised for 24 hours, whereas in EHI pigs rectal temperature decreased faster (within 12 hours) (Fig 6).In control groups, overall temperatures were on average 0.16°C higher in BHC pigs as compared to EHC pigs (p = 0.002).

BHI and EHI pigs showed a lower growth rate (p<0.001) in the period post infection with PRRSV (BHI: 538.8 ± 48.3 g/day; EHI: 594.9 ± 49.3 g/day) as compared to control groups (BHC: 714.2 ± 33.7 g/day; EHC: 715.3 ± 35.0 g/day). Growth rate in the period after *A*. *pleuropneumoniae* infection was also lower in the infected pigs (BHI: pigs 371 ± 29.8 g/day vs. BHC: pigs 725 ± 79.4 g/day and EHI pigs: 447.6 ± 43.9 g/day vs. EHC pigs: 775 ± 64.6g/day; p<0.001).

### White blood cell counts

**Fig 7:**
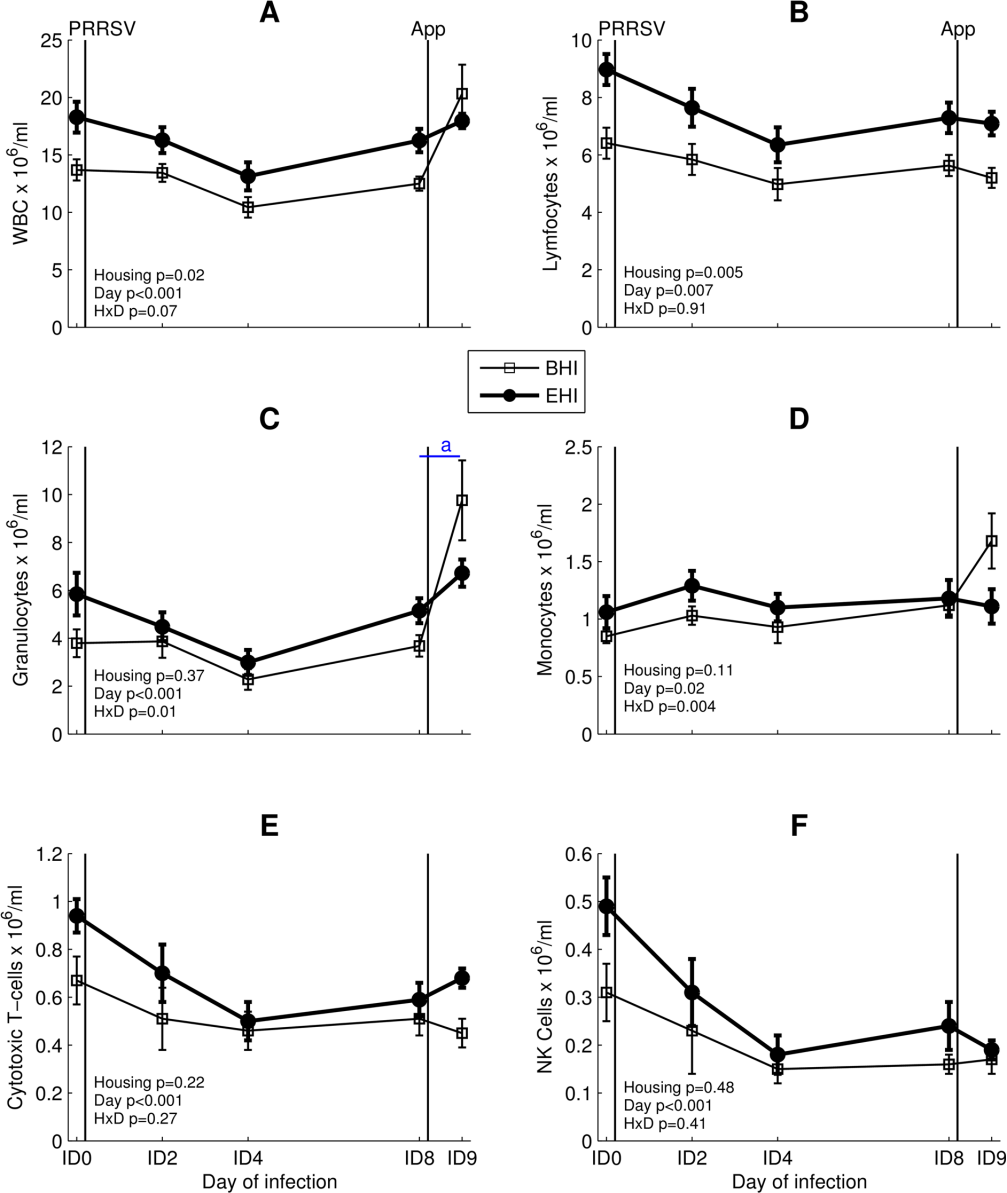
Absolute numbers of blood cells counts in BHI and EHI pigs (mean ± standard error). (A) WBC. (B) Lymphocytes. (C) Granulocytes. (D) Monocytes. (E) Cytotoxic T-cells. (F) NK cells. Moments of infections are represented as black vertical lines. HxD: housing-day interaction. ‘a’ denotes a significant increase between ID8 and ID9 in BHI pigs (post hoc comparison, p<0.05).

Total white blood cell (WBC) and lymphocyte counts were significantly elevated in serum of EHI pigs as compared to BHI pigs, but this overall housing effect was not observed in the other cell populations (Fig 7, results of memory and naive T-cells not shown). All cell populations showed an overall day effect. For granulocytes, monocytes and memory T-cells an interaction effect between housing and day was also found. Post hoc pairwise comparisons showed that granulocyte counts significantly increased in BHI pigs between ID8 and ID9 (p<0.001, Fig 7C), but not in EHI pigs.

### Broncho alveolar cell populations

Housing profoundly affected cellular composition of the BALF in infected pigs. A rise in the percentage of granulocytes (1.7 fold) and T-lymphocytes (6 fold) in lung fluid was measured in both infected groups (BHI and EHI pigs) as compared to their respective control groups (BHC and EHC, Table 4). Especially the relative amount of CD8^+^CD4^-^ (cytotoxic T–cells, 11.5 fold) and CD4^+^CD8^+^ (activated/memory T-cells, 8.7 fold) increased as compared to control pigs, whereas a relative decrease of CD14^+^ macrophages (1.2 fold) was observed (Table 4). Also a significant change in the expression (MFI) of the phenotypic markers CD14 (up), CD163 (down) and TLR4^+^ (down) was measured after infection in both groups (Table 4).

**Table 4:** Phenotypic markers of BALF Cells (mean percentages and MFI ± standard error).

Cell types	Phenotype	BHI	EHI	BHC	EHC	Infection effect
		%	%	%	%	
*Granulocytes*	Granulocytes	2.45±0.54	2.44±0.36	1.42±0.29	1.49±0.22	0.004
*Lymphocytes*						
T cellsT-Helper Cytotoxic T	CD3^+^	11.87±1.74	11.51±1.08	2.15±0.18	1.78±0.26	<0.001
	CD4^+^CD8^-^	2.76±0.48	2.49±0.26	1.21±0.13	0.85±0.12	<0.001
	CD8^+^CD4^-^	7.53±1.62	7.43±0.84	0.78±0.08	0.52±0.06	<0.001
Memory T	CD4^+^CD8^+^	0.3±0.07	0.22±0.03	0.04±0.01	0.02±0	<0.001
NK	CD3^-^CD8^+^	0.21±0.04	0.19±0.03	0.08±0.01	0.05±0.01	<0.001
*Macrophages*	%	%	%	%	%	
	CD14^+^	67.76±4.21	74.76±2.72	89.38±0.92	88.56±0.97	<0.001
	CD172a^+^	68.7±4.21	74.96±2.72	88.7±0.92	88.14±0.89	<0.001
	SLA II-DR^+^	63.62±5.01	68.89±3.21	88.63±0.88	88.39±0.86	<0.001
	CD163^+^	56.46±5.97	65.06±2.87	87.34±0.94	86.38±0.95	<0.001
	TLR4^+^	51.27±4.48	47.75±2.76	62.07±3.48	50.8±2.73	0.05
	CD172a^+^/TLR4^+^	41.83±4.31	37.97±2.15	54.98±6.56	42.74±2.35	0.01
*Macrophages*	MFI	MFI	MFI	MFI	MFI	
	CD14^+^	165.22±10.74	172.15±6.38	150.34±6.69	143.67±4.29	0.01
	CD172a^+^	377.44±41.34	325.22±17.36	400.72±35.06	355.41±13.19	0.30
	SLA II-DR^+^	274.74±25.36	263.15±26.23	339.34±47.47	284.96±16.04	0.10
	CD163^+^	750.32±82.29	775.58±59.62	940.35±66.42	895.18±68.8	0.01
	TLR4^+^	456.79±51.45	452.3±48.25	738.73±77.88	707.51±62.54	<0.001

MFI: Mean fluorescence intensity; BALF: Bronchoalveolar lavage fluid

An overall housing effect, was seen for CD172a^+^/TLR4^+^ macrophages (p = 0.02). Analysis of housing effect on infected and control animals separately showed that BHC pigs had higher percentages of TLR4^+^ and CD172a^+^/TLR4^+^ macrophages as compared to EHC pigs (Table 4, p = 0.02 and p = 0.01 respectively).

## Discussion

The use of environmental enrichment has repeatedly been proposed to enhance welfare of farm animals [6, 8, 21]. Our study confirms a decrease of stress related behaviour and decreased skin lesion scores in pigs provided with enrichment materials, indicating improved welfare, and simultaneously shows that enriched housing significantly reduces disease susceptibility to co-infection with PRRSV and *A*. *pleuropneumoniea*, expressed as a reduction of clinical and histo-pathological signs, of which the eight-fold difference in percentage of pigs with lung lesions was the most remarkable. Most studies on the relation between stress and occurrence of disease symptoms, use stress enhancing conditions, such as heat or cold stress, crowding, forced exercise, social stress, transportation, restraint/immobilization, avoidance learning, isolation or other stressful (often short term) life events [1]. In this study we used social and environmental enrichment, applied from birth onwards, to enduringly facilitate the development and display of natural (social) behaviour in contrast to ‘conventional husbandry systems conditions’ where behavioural needs of pigs are poorly met.

In our experiment we ensured a constant faecal pathogenic load by removing faeces from all pens daily. Also contamination with other pathogens was excluded as all materials that served as enrichment were replenished daily and were made germ free by γ-irradiation. So despite the same infectious doses of both pathogens, pigs in the enriched housing regime were better in preventing clinical manifestation of co-infection, resulting in profoundly less pigs with lung lesions and less severe pathologic tissue damage in the lungs, as compared to BHI pigs. The more severe histo-pathological damage in the lungs of BHI pigs also coincided with the more pronounced clinical read outs in these pigs in terms of rectal temperature, sickness behaviour as well as the more pronounced inflammatory reaction of different blood cell counts after *A*. *pleuropneumoniae* infection. In the BALF the relative increase in granulocyte and lymphocyte populations and decrease in macrophages after infection was similar in both BHI and EHI pigs, indicating that housing did not cause differences in immunological responses within the right cranial lung lobe, in which we did not detect typical *A*. *pleuropneumoniae* lesions.

Another remarkable finding was the faster viral clearance in EHI pigs as compared to BHI pigs. Both BHI and EHI pigs showed a similar rise of viral RNA in serum 4 days after infection, which is conform other studies in pigs infected with PRRSV serotype 1 LV-Ter Huurne strains [51]. This indicates that PRRSV infection initially affected both groups similarly. The viral RNA serum curve of BHI pigs followed those earlier described [51], whereas EHI pigs were able to reduce viral RNA in serum faster, with a statistically lower level 8 days after PRRSV infection. So, at the moment of *A*. *pleuropneumoniae* infection BHI pigs still had higher viral serum levels of PRRSV, whereas in EHI pigs it was already reduced. The inflammatory impact of PRRSV in the lungs of BHI pigs was also higher. PRRSV infection in lungs is histologically characterized by septal thickening by macrophages and perivascular cuffing [53, 54]. BHI pigs showed higher peri-bronchiolar and peri-vascular infiltrates scores as compared to EHI pigs post mortally, representing the more effective containment of PRRSV infection by the EHI pigs, which did however not result in differences in temperature level or sickness behaviour the period between PRRSV and *A*. *pleuropneumoniae* infection (ID1- ID8).

PRRSV is supposed to dampen both innate and specific immune responses as infection with PRRSV alters cytokine production of macrophages and monocytes derived dendritic cells, and modifies the expression of molecules involved in antigen presentation [34, 54, 55]. As a consequence, NK cell activation and mobilization of cells from the acquired arm of the immune system are delayed [34]. The faster clearance of PRRSV in EHI pigs could have diminished the impact of the secondary A. *pleuropneumoniae* infection that followed at ID8 in our experiment.

Moreover, we found overall higher levels of WBC as well as higher levels of lymphocytes in EHI pigs as compared to BHI pigs. Deep straw bedding has previously been found to influence variables related to (innate) immunity as well as to stress physiology as compared to relatively barren housing [33]. Differences in immune related variables possibly influence health status and responses to infections in pigs. In contrast to our results, the enriched housed pigs in this previous study had lower WBC than barren housed pigs [33]. A possible explanation for the contrasting outcome as compared to the previous study may relate to differences in starting point and duration of the enrichment provided, the age at which the animals were tested and the type of enrichment used. In our study, difference in rearing of BH and EH pigs started directly after birth, and included also social enrichment, i.e. mixing with another litter early in life. We do not consider the overall higher WBC level as the result of infection, but we cannot exclude that the EHI pigs have been challenged with environmental microbes, although all enrichment materials had been decontaminated. Possible explanations for our found differences in overall levels and responses after infections of the white blood cell counts remain unknown at this point.

Cytotoxic T-cells and NK cells are considered to play a role in viral clearing [58]. It is suggested that enhanced immune responses after PRRSV infection may lead to more severe clinical disease and gross pathology, but also supports an enhanced viral clearance [51, 58–60]. We did however not find differences in clinical findings or immunological responses between the barren housed pigs and enriched housed pigs directly after PRRSV (ID1 until ID8).

The relative decrease of lung macrophages in BALF in both EHI and BHI groups is probably due to influx of lymphocytes and granulocytes, but is also in line with the often proposed destructive effect of PRRSV on lung macrophages. It is suggested that this destructive effect of macrophages would make the lungs more susceptible for secondary infections [61].

Barren housed control pigs showed higher percentages of TLR4^+^ and CD172a^+^/TLR4^+^ macrophages as compared to EHC pigs. The control animals refer to differences in housing regime without infection, or to the situation prior to infection. TLR4^+^ is an essential part of the LPS (lipopolysaccharide) receptor binding complex [62]. Other components of this complex are CD14 and LPS binding protein (LBP). TLR4^+^ is stimulated by LPS from gram-negative bacteria and fusion (F) protein from respiratory syncitial virus (RSV) [63] and binds to the earlier formed CD14-LPS complex [64]. Stimulation of TLR4^+^ can induce potent responses such as sepsis and inflammation induced damage by release of pro inflammatory cytokines. The lower percentage of macrophages with TLR4^+^ markers in EHC pigs, may be related to a lower sensitivity of alveolar macrophages for LPS in the EH pigs as compared to BH pigs. This may be of interest when studying the complex pathways that lie behind the different clinical responses to co-infection of the two housing regimes.

Our behavioural observation demonstrate that a combination of rooting substrate, social enrichment and a larger pen size decreases stress related behaviour such as mounting behaviour and oral manipulations directed at pen mates and the pen itself, which is in line with our expectations and as described in earlier studies [6,19,54,57–61]. Although the occurrence of manipulative activities directed at pen mates is mostly considered to reflect a limitation in performing normal explorative behaviours such as rooting, chewing on substrate or foraging behaviour [54,57,61], it might also be considered as a strategy to cope with stress [62]. Behavioural differences between BH and EH pigs in this study were also indicated by the skin lesion scores. Engagement in reciprocal fighting has previously been found to result in lesions to the anterior third of the body, whilst the receipt of bullying (e.g. unilateral fighting) leads to lesions accruing on the caudal third of the body [65]. Overall higher mounting and manipulation of other pigs in barren housed pigs were seen, and higher skin lesions scores especially at the middle and rear of the body after weaning, indicating more bullying [53]. The smaller pen size might have inhibited retreat of the bullied pigs (limited possibilities to flee to prevent hierarchy battles), causing more skin lesions in BH pigs, especially at the middle and back. EH pigs in our study might also have developed better social skills, such as a more adequate reaction to threat, more flexibility in using aggression in conflicts and the development of submissive behaviours [66] during the pre-weaning mixing period as compared to BH pigs that were confronted with totally new pigs after weaning. Other studies have demonstrated that the behavioural differences between pigs in barren and enriched housing conditions are also reflected in stress physiology measurements, such as HPA-axis (re)activity [9, 11, 33], indicating that BH pigs were probably also more (stress) physiologically challenged.

In our study, the enrichment stimulated the EH pigs psychologically differently as compared to the barren housed pigs and diminished (chronic) stress in the animals. Chronic stress in general is considered a potential influencing factor on disease susceptibility, however the complex pathways that mediate the effects of stress on infectious diseases, are not completely understood [1]. The better psycho-physiological and immunological state of the EH pigs likely positively affected their immune and inflammatory responses [67–71], and in this way, diminished the clinical manifestation. Our results are also in line with the increasing epidemiological evidence in humans and other species that environmentally induced adaptations, occurring at crucial stages of life, can potentially change behaviour, disease susceptibility and survival also known as the ‘early origins of the adult disease susceptibility’ hypothesis [4, 72].

In conclusion, enriched rearing leads to a less severe onset and outcome of a PRRSV *A*. *pleuropneumoniae* co-infection. The enriched housed pigs showed a remarkably reduced impact of infection and were less prone to develop clinical signs of disease. We found more support for implementation of psychoneuro-immunological intervention strategies to reduce the impact of infectious diseases and by this reducing antibiotics use. Future research should investigate the possible involved explanatory pathophysiological pathways.

## Abbreviations

PRRSV: porcine reproductive and respiratory syndrome virus
*A*. *pleuropneumoniae*: *Actinobacillus pleuropneumoniae*
PRDC: Porcine Respiratory Diseases Complex
HPA axis: hypothalamic-pituitary-adrenal-axis
BH: Barren Housed
EH: Enriched Housed
BHI: Barren Housed Infected
EHI: Enriched Housed Infected
ID1 t/m ID11: Infection day
HEPA: High Efficiency Particulate Air
SB plate: Sheep blood agar plate
NAD plate: nicotinamide adenine dinucleotide (NAD) plate
CFU: Colony forming units
BALF: Bronchoalveolar lavage fluid
HE: Haematoxylin-Eosin
RNA: Ribonucleic Acid
PCR: Polymerase chain reaction
TCID50: Tissue culture infectious dose
EDTA: Ethylenediaminetetraacetic acid
WBC: White Blood Cell
FCM: Flow Cytometry
FACS: Fluorescence-activated cell sorter
FCV: Forward scatter
SSC: Sideward scatter
NK: Natural killer cell
MFI: Mean fluorescence intensity
CD: Cluster of differentiation
mAb: Monoclonal antibody
SLA: Swine leucocyte antigen
TLR: Toll like receptor
GLM: General Linear model
SEM: Standard error of the mean
LPS: Lipopolisaccharide
LBP: LPS binding protein
